# High success and low mortality rates with non-invasive ventilation in influenza A H1N1 patients in a tertiary hospital

**DOI:** 10.1186/1756-0500-4-375

**Published:** 2011-09-28

**Authors:** Karina T Timenetsky, Silvia HCT Aquino, Cilene Saghabi, Corinne Taniguchi, Claudia V Silvia, Luci Correa, Alexandre R Marra, Raquel AC Eid, Oscar FP dos Santos

**Affiliations:** 1 Critically Ill Patients Department, Albert Einstein Jewish Hospital (Avenida Albert Einstein, 627), Sao Paulo (zip code 05652900), Brazil

**Keywords:** influenza A H1N1, non-invasive ventilation, mortality, respiratory illness

## Abstract

**Background:**

In 2009, an outbreak of respiratory illness caused by influenza A H1N1 virus occurred worldwide. Some patients required Intensive Care Unit (ICU) admission. The use of non-invasive ventilation (NIV) in these patients is controversial, as the aerosol dispersion may contaminate the environment and health-care co-workers.

**Methods:**

Describe the respiratory profile, the mortality rate, and the benefit of using NIV in patients with confirmed diagnosis of influenza AH1N1 who were admitted in the ICU during the year 2009.

**Results:**

A total of 1, 401 cases of influenza A H1N1 were confirmed in our hospital by real-time RT-PCR in 2009, and 20 patients were admitted to the ICU. The patients' ages ranged from 18 to 74 years (median of 42). Acute Respiratory Failure (ARF) was present in 70% of patients. The median Acute Physiology and Chronic Health Evaluation II score was 7 (range 7 to 25). Of the 14 patients who developed ARF, 85.7% needed NIV and 14% needed invasive MV at admission. Our success rate (41.6%) with NIV was higher than that described by others. The hospital mortality rate was 2.1%. When influenza A H1N1 arrived in Brazil, the disease was already on endemic alert in other countries. The population was already aware of the symptoms and the health-care system of the treatment. This allowed patients to be properly and promptly treated for influenza A H1N1, while health-care workers took protective measures to avoid contamination.

**Conclusion:**

In our study we found a high success and low mortality rates with non-invasive ventilation in patients with influenza A H1N1.

## Background

In late March of 2009, an outbreak of respiratory illness caused by swine-origin influenza A H1N1 virus occurred in Mexico. All patients developed acute respiratory distress [1], of which 38% died. This pandemic disease spread quickly throughout the world [1-8].

Some individuals with influenza A H1N1 develop severe forms of the disease that require hospitalization in the intensive care unit (ICU), mostly due to acute respiratory failure (ARF). Different studies have reported that between 62 and 100% of the patients require mechanical ventilation (MV), with a mortality rate varying from16 to 58% [1-5,7-9].

Although frequently used, invasive MV has several complications, such as ventilator-associated pneumonia, which is a risk factor for mortality and for diaphragmatic dysfunction, which may lead to diaphragm myofiber atrophy [9-11].

Only two studies have addressed the issue of using noninvasive ventilation (NIV) in patients with influenza A H1N1 virus, but the controversies regarding its benefit are still unresolved [2,8]. There is a concern that NIV might lead to aerosol dispersion, which may contaminate health-care co-workers [2,8,12]. On the other hand, NIV prevents the need for endothracheal intubation in patients with ARF, minimizing related complications [9,12,13].

In our hospital we had a total of 1, 401 confirmed cases of influenza A H1N1. In this study we describe the respiratory profile, the mortality rate, and the benefit of using NIV in patients with confirmed diagnosis of influenza AH1N1 who were admitted in the ICU during the year 2009.

## Methods

We retrospectively evaluated the medical charts and laboratory exams of all influenza A H1N1 virus-suspected patients who were admitted to the ICU during the year of 2009.

This study was conducted in a 38-bed medical-surgical ICU of a tertiary-care private hospital in Sao Paulo, Brazil. This is an open-staffing-model ICU where approximately 2, 200 patients are admitted annually. We have a nurse:bed ratio of 1:4 in our ICU. This study was approved by the ethics committee of the Albert Einstein Jewish Hospital and patient's consent was waved due to the study design.

To confirm the cases of swine-origin influenza A H1N1 virus by real-time RT-PCR (RT-RTPCR), nasopharyngeal-swab samples were collected at hospital admission and respiratory secretions obtained from intubated patients. RT-RTPCR testing was performed in all patients with suspected signs of influenza A H1N1 that were treated at our hospital according to published guidelines from the Center for Disease Control (CDC) [14,15].

All influenza A H1N1 virus-confirmed patients admitted to the ICU were included in the study.

We also collected demographic data and information on coexisting medical conditions, microbiology findings, incidence of ARF, the requirement of MV (whether invasive or noninvasive at ICU admission and during ICU stay), laboratory findings, and gas exchange ratio at admission (PaO_2_/FiO_2_), for each patient. For those patients who required invasive MV, we evaluated the need for alveolar recruitment maneuver, MV time, and MV weaning success. To determine the severity of illness, the Acute Physiology and Chronic Health Evaluation (APACHE) II score was determined in all patients within 24 hours of admission in the ICU. We also collected information on the length of the entire hospital stay (including the time in the ICU) and on mortality rate.

MV weaning success was considered when the patient was able to tolerate at least 48 hours without invasive mechanical ventilation.

Noninvasive mechanical ventilation was instituted in the confirmed influenza A H1N1 when there were signs of acute respiratory failure at hospital admission or during ICU stay. The signs of acute respiratory failure were tachypnea (respiratory rate higher than 35 rpm), hypoxemia (PaO2 < 80 mmHg), use of accessory respiratory muscles, and need of high oxygen concentration (higher than 40% delivered by simple facial mask or using mask with a non re-breathing system). NIV success was considered when patient was able to improve oxygenation, respiratory rate (lower than 35 rpm), carbon dioxide concentration, the use of accessory respiratory muscles within 2 hours of NIV. In case this improvement was not achieved or patient did not tolerate the use of NIV, they were promptly intubated and assisted with invasive mechanical ventilation. Patients were informed of the reasons to use NIV to treat acute respiratory failure, or the need to initiate invasive mechanical ventilation. Patients needed to collaborate with NIV to use it, being alert and responsive to commands without agitation.

NIV was delivered by a specific ventilator, BiPap^® ^Vision^® ^(Philips - Respironics) providing a pressure support ventilation through a total face mask. The respiratory therapists were responsible for this therapeutic intervention. Our ICU has a respiratory therapists:bed ratio of 1:5.

Patients that were admitted with signs of acute respiratory failure as described above, with extreme levels of hypoxemia (PaO2 lower than 60 mmHg with a high oxygen concentration delivered by a mask with a non re-breathing system - 100%), low level of consciousness, or refuse to use NIV were promptly intubated at ICU admission.

MV weaning success was considered when the patient was able to tolerate at least 48 hours without invasive mechanical ventilation.

### Statistical Analysis

Categorical variables were expressed as absolute and relative frequencies (percentages). The quantitative variables were expressed as mean and standard deviation if normally distributed, or as median and interquartile range if distributed otherwise. The software R, version 10.1 was used to compute the descriptive statistics [16].

## Results

During the year of 2009, we had a total of 4, 308 suspected cases of influenza A H1N1 that were treated in our hospital. Of these, 1, 401 patients had confirmed diagnosis of influenza A H1N1 and only 139 of these patients needed hospital admission. There were 86 patients admitted to the ICU with suspected cases of swine-origin influenza A H1N1 virus. Of these cases, 20 patients were confirmed with RT-RTPCR test. Our analyses were based only on the 20 patients with confirmed influenza A H1N1 that were admitted to the ICU. All patients with suspect signs of influenza A H1N1 admitted to our hospital were treated with oseltamivir within 48 hours of symptoms initiation.

The characteristics of the 20 patients admitted to the ICU with confirmed cases of influenza A H1N1 virus are listed in Table 1. The patients' ages ranged from 18 to 74 years (mean of 42.6 years). Eleven patients (55%) were male. Ten patients had coexisting medical conditions as follows: asthma (in two patients), diabetes mellitus (in three patients, two of whom were also transplanted patients), coronary insufficiency (in two patients, one of whom had also chronic obstructive pulmonary disease), chronic obstructive pulmonary disease (in one patient), renal transplant (in two patients), liver transplant (in one patient), and non-Hodgkin's lymphoma (in one patient).

**Table 1:** Characteristics of the 20 patients with confirmed cases of swine-origin influenza A H1N1 virus admitted to the ICU in 2009.

Variables	Values
**Age **- years	
Mean (95% CI)	42.6 (34.7-50.3)
**Male gender **- n (%)	11 (55%)
**Coexisting condition **- n (%)	
Asthma	2 (10%)
Diabetes mellitus	3 (15%)
Coronary insufficiency	2 (10%)
Chronic Obstructive Pulmonary Disease (COPD)	1 (5%)
Renal transplant	2 (10%)
Liver transplant	1 (5%)
Non-Hodgkin's Lymphoma	1 (5%)

95% CI (confidence of interval)

As shown in Table 2, ARF was present in 14 patients (70%), due mainly to pneumonia (9 patients, 64.3%) and also to acute respiratory distress syndrome (ARDS; 5 patients, 35.7%), none of the patients presented acute hypercapnic respiratory failure. The APACHE II score ranged from 7 to 25 (median of 7). Gas exchange ratio (PaO_2_/FiO_2_) at hospital admission ranged from 59 to 361 (median, 226.5).

**Table 2:** Characteristics of the 20 study patients who had confirmed infection with Novel Swine-Origin Influenza A (H1N1) Virus admitted to the ICU in 2009.

Variables	Values
**APACHE II score**	7 (7-12)
Median (IQR)	
**Presence of Acute Respiratory Failure (ARF) **- n (%)	14 (70%)
**Causes of Acute Respiratory Failure (ARF) **- n (%)	
Acute Respiratory Distress Syndrome (ARDS)	5 (35.7%)
Pneumonia	9 (64.3%)
**PaO**_**2**_**/FiO**_**2 **_**ratio**	
median (IQR)	226.5 (106-315)
**Mechanical Ventilation** (**MV) on admission **- n (%)	
No	6 (30%)
Non-Invasive Ventilation (NIV)	12 (60%)
Invasive Mechanical Ventilation (MV)	2 (10%)
**Non-Invasive Ventilation (NIV) success rate **- n (%)	5 (41.6%)
**Invasive Mechanical Ventilation (MV) during ICU **- n (%)	9 (45%)
**Mechanical Ventilation (MV) time**	
Mean (95% CI)	6 days (4.2-9)
**Recruitment maneuver **- n (%)	5 (55%)
**Successful Mechanical Ventilation (MV) weaning rate **- n (%)	8 (88.8%)
**ICU length of stay**	
Median (IQR)	4 days (2-6)
**Hospital length of stay**	
Median (IQR)	10 days (3-18)
**ICU mortality rate **- n (%)	3 (15%)
**Hospital mortality - **n (%)	3 (2.1%)

95% CI (confidence interval); IQR (interquartile range)

Of the 14 patients who developed ARF, 12 (85.7%) needed NIV and 2 (14%) needed invasive MV at admission. The number of days on NIV ranged from 1 to 11 (median of 3 days), being successful in 5 patients (41.6%; Table 2), none of them had hypercapnic respiratory failure. The median carbon dioxide arterial pressure (PaCO_2_) before using NIV was 33.8 mmHg (range of 21 to 45 mmHg) and after 2 hours of NIV was 35.8 mmHg (range of 30 to 43 mmHg). The remaining patients had to be intubated. We had no ICU health-care co-workers contaminated by influenza A H1N1 during the study period, the contamination was monitored by the hospital infection control department, evaluating all health-care co-workers that were treating these patients with RT-RTPCR testing. There were no signs of skin breakdown with the use of total face mask to deliver NIV and all these patients cooperated with NIV.

Invasive MV was required in 2 patients at admission and in 7 after NIV during hospitalization, with a total of 9 patients undergoing invasive MV. Of these 9 patients, we implemented alveolar recruitment maneuver in 5 patients, but none of them responded successfully, and 8 (88.8%) were successfully weaned from MV. Invasive MV time ranged from 1 to 12 days (median, 6 days; Table 2).

ICU length of stay ranged from 1 to 14 days (median, 4 days) and hospital length of stay ranged from 1 to 56 days (median, 10 days). The ICU mortality rate was 15% (3 patients) and hospital mortality rate was 2.1% for the confirmed cases of influenza A H1N1 virus.

The 3 patients who died in the ICU had a coexisting medical condition. One patient had arterial hypertension, the second was a transplanted patient, and the third had coronary insufficiency.

## Discussion

In this study we identified all patients with confirmed diagnosis of influenza A H1N1 admitted to the Albert Einstein Jewish Hospital during the year of 2009. We identified 1, 401 patients with the infection and who were treated in our hospital. Of these, only 139 (9.9%) patients needed hospitalization. Of these 139 patients, 20 (14.3%) were admitted to the ICU. Our ICU admission incidence was lower than that reported in most studies [3,9]. The exception was a study done in Canada that reported an even lower incidence (8.1%) [8].

Cases of acute respiratory failure due to influenza A H1N1 affecting patients younger than the expected age for patients with seasonal influenza have been previously reported [1-8]. Our findings are consistent with these reports.

In regard to the severity of illness, our APACHE II score (7) was lower than that of other studies, which reported a median score around 13 to 19 [1,2,8]. Our APACHE II score was probably lower than other studies because the disease was already an endemic disease when it reached Brazil and the population and health-care co-workers were already alert to the disease signs and received early treatment. This is probably also the reason for our low number of patients admitted to the ICU as reported by other studies.

Similar to other studies, our study identified chronic lung disease as the most frequent coexisting medical condition [1-3,5,8,9]. On the other hand, a study by Cao et al. described arterial hypertension as the most frequent coexisting medical condition [6].

Most of our patients had ARF, as seen in other studies, with the exception of one study from Japan that did not report any case of ARF between May and June of 2009 [1-3,8,9]. In our study, we had 85.7% of the patients who used NIV at ICU admission to treat ARF. Our incidence was higher than that of the other two studies that mentioned the use of NIV at ICU admission (around 33%) [2,8]. In this study we had a success rate with NIV in 41.6% of the patients, showing greater success with NIV when compared with the other two studies (25% in Spain [2] and14.6% in Canada [8]).

We believe that this difference in NIV success is due to the disease severity, because our APACHE II score was lower than that described by others [2,8]. Because of the low NIV success rate and high endotracheal intubation incidence previously reported in several studies, many researchers stated that NIV should not be routinely used in patients with pandemic respiratory infections [1,2,8,17].

Many researchers are concerned about using NIV during pandemic respiratory infections due to the infectious exhaled aerosol that can reach the environment through the exhalation ports in the NIV mask or tubing, which may contaminate health-care co-workers [17,18]. For instance, in a recent study from Australia [9] in which a significant correlation with the need for invasive MV and mortality rates was observed (Odds ratio 5.51; 95% CI 3.95-9.94; p < 0.001), NIV was never considered as an option.

In the study by Perez-Padilla et al. [1], the authors reported 22 (11.5%) cases of health-care co-worker contamination with influenza A H1N1, while treating the first 3 patients who were admitted to the hospital. None of these patients were undergoing NIV. After an infection-control measure that enforced patient isolation in specific hospital areas, use of N95 respirators in addition to goggles, gowns, and gloves, and constant use of gel-alcohol hand sanitizer, no more health-care co-workers were contaminated by the disease.

This was not the case in our hospital, where our NIV success rate was higher than everywhere else, and no health-care co-worker was contaminated through NIV and all health-care co-workers used infection control measures to avoid contamination, such use of goggles, gowns, gloves, and use of gel-alcohol hand sanitizer. All influenza A H1N1 ICU patients stayed in individual and isolated hospital bed, to avoid contamination to other patients. Our success rate with NIV indicates that patients with influenza A H1N1 can benefit from it, thus preventing the need for invasive MV, and minimizing the incidence of complications such as ventilator-associated pneumonia [11]. This was the situation described in Mexico [1], where 4 (22%) patients underwent invasive MV and had ventilator-associated pneumonia.

Cheung et al. [17] reported their experience with 20 patients undergoing NIV during severe acute respiratory syndrome (SARS) in 2003 at a Hong Kong hospital. To reduce the risk of contamination the staff used a viral/bacterial filter, N95 respirators, and an oronasal mask to prevent large leaks through the mouth and exhalation valve. They were able to prevent endotracheal intubation in 14 patients (70%) and none of the health-care co-workers were contaminated. In our hospital all the protective measures were used to prevent contamination of health-care co-workers and other ICU patients.

The fact that influenza A H1N1 arrived in Brazil after being already an endemic alert in other countries provided the necessary time for the government to set strategies to control its spread. Additionally, the population was already aware of the symptoms and the health-care system of the treatment. This allowed patients to be properly and promptly treated for influenza A H1N1, while health-care workers took protective measures to avoid contamination.

In this scenario, NIV can be safely used in patients with influenza A H1N1. Nevertheless, the same indication for using NIV in patients requiring MV for ARF should be considered for patients with influenza A H1N1.

In regard to invasive MV, our study had a lower incidence at admission, when compared to other studies [1-3,8,9]. This can also be related to our low APACHE II score, as the study by Rello et al. [2] described that patients who failed NIV had a higher APACHE II score than those who were successful. Our mechanical ventilation time was lower than that described by others [1,2,8,9].

In our study 5 patients developed ARDS and were intubated. Of these patients, all underwent alveolar recruitment maneuver with high end-expiratory pressure levels to improve oxygenation, without any related complications. In the studies on influenza A H1N1 patients currently available [1-9], none mention using this maneuver to improve oxygenation. In one study, Kumar et al. [8] reported using inhaled nitric oxide, high frequency oscillatory ventilation, and extracorporeal membrane oxygenation and had 8.3% incidence of barotraumas.

Regarding the ICU length of stay, our findings were lower than those reported in previous studies [8,9]. However, in regard to our hospital length of stay, our findings were similar to those from a study from Australia [9].

So far, patients with influenza A H1N1 admitted to hospitals worldwide had a high mortality rate (13 to 39%) [1-9]. However, in our study the hospital mortality rate was 2.1%. Beside the low APACHE II score of our patients, it is possible that our low mortality rate is also associated with a lower number of patients undergoing invasive mechanical ventilation, as compared to other studies [1-4,8,9].

## Conclusion

In our study we found a high success and low mortality rates with non-invasive ventilation in patients with influenza A H1N1. Although our study is limited to one center only and cannot be representative of the scenario in Brazil, we had 1, 401 patients with confirmed influenza A H1N1, of whom most were successfully treated in our hospital as described.

## Competing interests

The authors declare that they have no competing interests.

## Authors' contributions

KTT participated in the study design, data collection, performed statistical analysis, and writing the manuscript. SHCTA participated in the study design and data collection. CS and CT participated in the data collection. CVS and LC were responsible for collecting the RT-RTPCR testing results and also helped with drafting the manuscript. ARM participated in the statistical analysis and in drafting the manuscript. RACE and OFPS participated in the study design and in drafting the manuscript. All authors read and approved the final manuscript.

## References

[B1] Perez-Padilla R Rosa-Zamboni D Leon SP Hernandez M Quinones-Falconi F Bautista E Ramirez-Venegas A Rojas-Serrano J Ormsby CE Corrales A Higuera A Mondragon E Cordova-Villalobos JA Pneumonia and respiratory failure from swine-origin influenza A (H1N1) in Mexico N Engl J Med 2009 361 680 9 10.1056/NEJMoa0904252 19564631

[B2] Rello J Rodriguez A Ibañes P Sócias L Cebrian J Marques A Guerrero J Ruiz-Santana S Marquez E Nogal-Saez FD Alvarez-Lema F Martinez S Ferrer M Avellanas M Granada R Maraví-Poma E Albert P Sierra R Vidaur L Ortiz P del Portillo IP Galván B León-Gil C Intensive care adult patients with severe respiratory failure caused by influenza A (H1N1)v in Spain http://ccforum.com/content/13/5/R148 10.1186/cc8044 PMC2784367 19747383

[B3] Jain S Kamimoto L Bramley AM Schmitz AM Benoit SR Louie J Sugerman DE Druckenmiller JK Ritger KA Chugh R Jasuja S Deutscher M Chen S Walker JD Duchin JS Lett S Soliva S Wells EV Swerdlow D Uyeki TM Fiore AE Olsen SJ Fry AM Bridges CB Finelli L Hospitalized patients with 2009 H1N1 influenza in the United States, April-June 2009 N Engl J Med 2009 361 1935 44 10.1056/NEJMoa0906695 19815859

[B4] Libster R Bugna J Coviello S Hijano DR Dunaiewsky M Reynoso N Cavalieri ML Guglielmo MC Areso MS Gilligan T Santucho F Cabral G Gregorio GL Moreno R Lutz MI Panigasi AL Saligari L Caballero MT Egües Almeida RM Gutierrez Meyer ME Neder MD Davenport MC Del Valle MP Santidrian VS Mosca G Garcia Domínguez M Alvarez L Landa P Pota A Pediatric hospitalizations associated with 2009 pandemic influenza A (H1N1) in Argentina N Engl J Med 2010 362 45 55 10.1056/NEJMoa0907673 20032320

[B5] Shimada T Gu Y Kamiya H Komiya N Odaira F Sunagawa T Takahashi H Toyokawa T Tsuchihashi Y Yasui Y Tada Y Okabe N Epidemiology of influenza A(H1N1)v virus infection in Japan, May-June 2009 Euro Surveill 2009 14 pii19244 10.2807/ese.14.24.19244-en 19555600

[B6] Cao B Li X-W Mao Y Wang J Lu HZ Chen YS Liang ZA Liang L Zhang SJ Zhang B Gu L Lu LH Wang DY Wang C Clinical features of the initial cases of 2009 pandemic influenza A (H1N1) virus infection in China N Engl J Med 2009 361 2507 17 10.1056/NEJMoa0906612 20007555

[B7] Hackett S Hill L Patel J Ratnaraja N Ifeyinwa A Farrogi M Nusgen U Debenham P Gandhi D Makwana N Smit E Welch S Clinical characteristics of paediatric H1N1 admissions in Birmingham, UK Lancet 2009 374 605 19700001 10.1016/S0140-6736(09)61511-7

[B8] Kumar A Zarychanski R Pinto R Cook DJ Marshall J Lacroix J Stelfox T Bagshaw S Choong K Lamontagne F Turgeon AF Lapinsky S Ahern SP Smith O Siddiqui F Jouvet P Khwaja K McIntyre L Menon K Hutchison J Hornstein D Joffe A Lauzier F Singh J Karachi T Wiebe K Olafson K Ramsey C Sharma S Dodek P Canadian Critical Care Trials Group H1N1 Collaborative Critically ill patients with 2009 influenza A (H1N1) infection in Canada JAMA 2009 302 17 1872 9 10.1001/jama.2009.1496 19822627

[B9] The ANZIC Influenza Investigators Clinical care services and 2009 H1N1 influenza in Australia and New Zealand N Engl J Med 2009 361 20 1925 1934 19815860 10.1056/NEJMoa0908481

[B10] Antonelli M Conti G Rocco M Bufi M De Blasi RA Vivino G Gasparetto A Meduri GU A comparison of noninvasive positive-pressure ventilation and conventional mechanical ventilation in patients with acute respiratory failure N Engl J Med 1998 339 7 429 435 10.1056/NEJM199808133390703 9700176

[B11] Rodrigues PM Carmo Neto E Santos LR Knibel MF Ventilator-associated pneumonia: epidemiology and impact on the clinical evolution of ICU patients J Bras Pneumol 2009 35 11 1084 91 10.1590/S1806-37132009001100005 20011843

[B12] Levine S Nguyen T Taylor N Friscia ME Budak MT Rothenberg P Zhu J Sachdeva R Sonnad S Kaiser LR Rubinstein NA Powers SK Shrager JB Rapid disuse atrophy of diaphragm fibers in mechanically ventilated humans N Engl J Med 2008 358 13 1327 1335 10.1056/NEJMoa070447 18367735

[B13] Keenan SP Mehta S Noninvasive ventilation for patients presenting with acute respiratory failure: the randomized controlled trials Respir Care 2009 54 1 116 26 19111111

[B14] CDC protocol of real-time RTPCR for influenza A (H1N1) Geneva: World Health Organization http://www.who.int/csr/resources/publications/swineflu/CDCRealtimeRTPCR_SwineH1Assay-2009_20090430.pdf

[B15] Pabbaraju K Wong S Wong AA Appleyard GD Chui L Pang XL Yanow SK Fonseca K Lee BE Fox JD Preiksaitis JK Design and validation of real-time reverse transcription-PCR assays for detection of pandemic (H1N1) 2009 virus J Clin Microbiol 2009 47 11 3454 3460 10.1128/JCM.01103-09 19726603 PMC2772599

[B16] R Development Core Team R: A language and environment for statistical computing R Foundation for Statistical Computing, Vienna, Austria http://www.R-project.org

[B17] Cheung TM Yam LY So LK Lau AC Poon E Kong BM Yung RW Effectiveness of noninvasive positive pressure ventilation in the treatment of acute respiratory failure in severe acute respiratory syndrome Chest 2004 126 3 845 850 10.1378/chest.126.3.845 15364765 PMC7094489

[B18] American Association for Respiratory Care AARC SARS guidance document 2003 http://www.aarc.org/resources/sars/sars%20aarc%20guidance%20document.pdf

